# Case Report: Differential diagnosis for tuberous sclerosis and neurofibromatosis type 1 diagnostic pitfall of aggressively enlarged right upper limb

**DOI:** 10.3389/fonc.2022.1007651

**Published:** 2022-11-24

**Authors:** Cheng-Jiang Wei, Li-Ling Peng, Man-Hon Chuang, Zhi-Chao Wang, Bin Wang

**Affiliations:** ^1^ Department of Plastic and Reconstructive Surgery, Shanghai Ninth People’s Hospital, Shanghai Jiao Tong University School of Medicine, Shanghai, China; ^2^ Shanghai Universal Medical Imaging Diagnostic Center, Huaxin Business Center, Shanghai, China

**Keywords:** tuberous sclerosis complex (TSC), neurofibromatosis type 1 (NF1), differential diagnosis, treatment, follow-up recommendation, case report

## Abstract

Tuberous sclerosis complex (TSC) is an inherited disorder that typically presents with seizures, developmental delay, cutaneous lesions, and facial angiomas. Clinical diagnosis of TSC based on symptoms is sometimes challenging due to its clinical similarities with neurofibromatosis type 1 (NF1), another type of neurogenetic tumor syndrome. Differential diagnosis should be carefully performed on the basis of clinical presentations, imaging, laboratory, and genetic testing. Here, we presented a case of a patient with an aggressively enlarged right upper limb in the NF1 clinic, who was initially suspected of a giant plexiform neurofibroma. However, differential diagnosis revealed TSC as the final diagnosis. The treatments for NF1 and TSC vary significantly, and misdiagnoses can lead to serious threat to the patients’ health. We also systematically reviewed all previous cases regarding differential diagnoses between NF1 and TSC. This case report can help clinicians make more accurate diagnoses and benefit the potential patient community.

## Introduction

**Figure 1:**
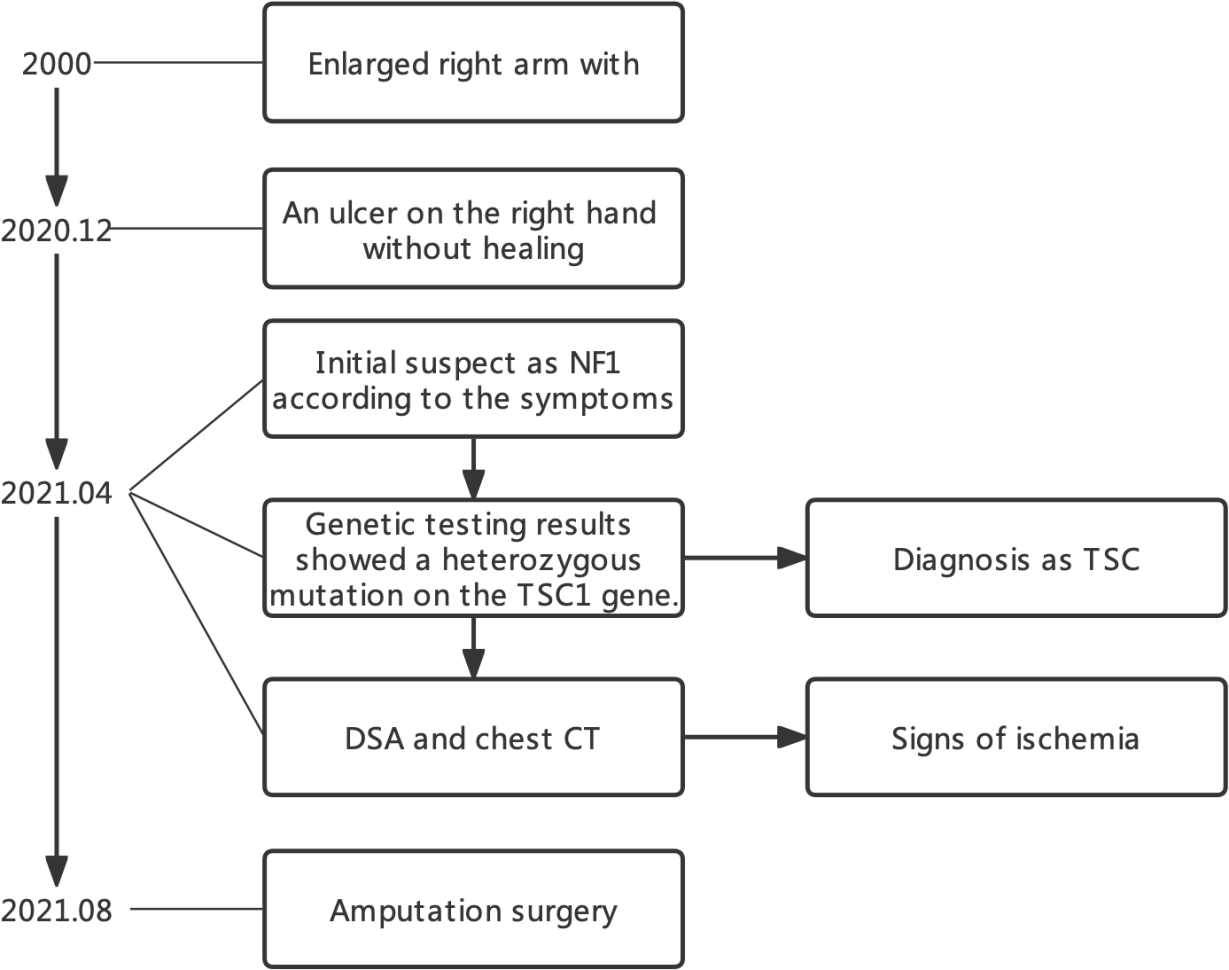
Timeline of this case.

Tuberous sclerosis complex (TSC) is a rare genetic disease characterized by seizures, developmental delay, and facial angiomas (Vogt’s triad) (1). Neurofibromatosis type 1 (NF1) is another neurogenetic tumor syndrome caused by the mutation of the *NF1* gene (2). There are similarities in clinical symptoms between TSC with NF1, which might cause misdiagnosis. Here, we described a case of a young patient with TSC with an enlarged right arm who was first diagnosed as NF1 during first visit to the clinic. Genetic testing revealed the *TSC1* mutation, and TSC was confirmed as the final diagnosis. Furthermore, the radiological imaging showed lung lymphangioleiomyoma and poor blood supply in the right arm. Last, we discussed the clinical treatment and follow-up recommendation for this patient (**Figure 1**).

## Case presentation

**Figure 2:**
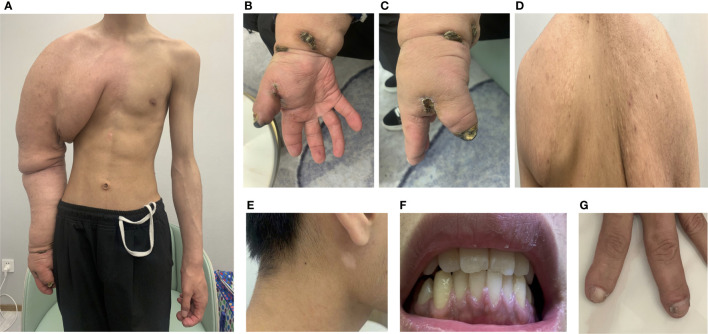
Clinical symptoms. **(A)** Enlarged right arm; **(B, C)** unhealing ulcer; **(D)** adenoma sebaceous; **(E)** depigmented macule; **(F)** gingiva; **(G)** periungal fibromas.

A 20-year-old man presented to our institution, complaining that the right upper limb was much thicker than the left from birth, along with the enlargement of his left index finger and middle finger (**Figures 2A–C**). The volume of the right upper limb increased significantly during puberty. Five months before this visit, the patient suffered from an ulcer on the right hand that had failed to heal. He did not pay much attention to the enlarged right arm as he has got used to the condition for over 20 years. The main complaint for this visit was the non-healing right-hand ulcer, which he thought could be solved after simple debridement. He showed little awareness and had limited knowledge regarding the disease. Upon questioning, the patient declared no history of seizures and showed no signs of amentia. His parents also reported that the patient had no history of autism.

On clinical examination, the right arm was significantly more prominent than the left, with the soft tissue being much thicker than the opposite side. Meanwhile, there were sebaceous adenomas on the back, depigmented macules on the face, and fibromas under the nail bed (periungual fibromas) and gingiva (**Figures 2D–G**). Other skin lesions of tuberous sclerosis such as ash-leaf spots and shagreen patches were not evident. The physical examinations of cardiovascular and central nervous systems were also normal.

**Figure 3:**
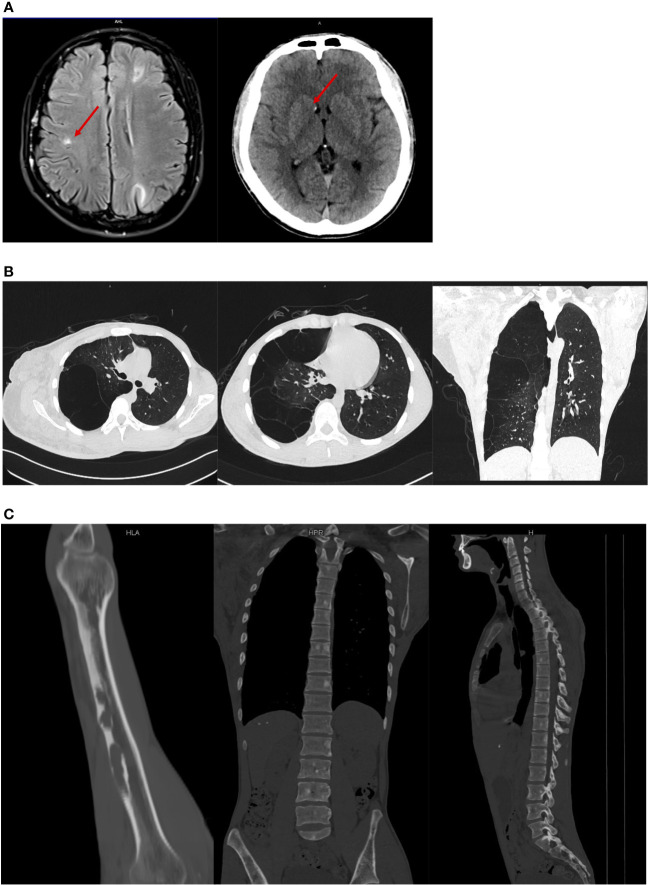
Medical imaging results. **(A)** Nodules showed on the head CT; **(B)** lymphangioleiomyomatosis on the chest CT and bone cysts in the different regions; **(C)** lymphangioleiomyomatosis on the chest CT and bone cysts in the different regions.

We initially suspected the case as plexiform neurofibroma with NF1 due to the enlargement of the right arm. However, the facial spots described by the patient as “café’ au lait spots” are actually depigmented macules. Whole-body imaging was then conducted for the patient. The head CT revealed subependymal, cortical, and subcortical nodules (**Figure 3A**). Meanwhile, we found lymphangioleiomyomatosis on the chest CT, focal sclerosis, and bone cysts in multiple bone regions such as the spine (**Figures 3B, C**). Although MRI might help clarify the nature of the enlarged right arm, it could not be conducted as the volume of the patient’s right arm exceeded the maximum size limit of our MRI machine. No hemorrhage and retinal hamartomas were found on ophthalmic examination, and mild mitral regurgitation was found on echocardiography.

Genetic testing was conducted for further differential diagnosis. No *NF1* mutation was found. Furthermore, there was a heterozygous mutation on the TSC1 gene.

On the basis of these features, a diagnosis of TSC was established, and the non-healing ulcer was considered to be associated with the vascular blockage caused by the thickened soft tissue. Digital subtraction angiography (DSA) was used to analyze the blood flow condition of the right arm (**Supplementary File 1**). The result showed insufficient blood supply in the distal right upper limb, and amputation appeared to be the only treatment plan for the patient. During consultation, the patient showed difficulties accepting this treatment plan and stated that he needed time for consideration. Approximately 4 months later, he finally accepted the amputation surgery of the right arm as the ulcer condition barely improved. After the surgery, we recommended life-long follow-up and monitoring of potentially fatal complications. Annual ophthalmology examination was also essential for the risk of hemorrhage. As the patient also presented with lung lymphangioleiomyoma and cyst, high-resolution CT should be performed every year and the annual pulmonary function examination was necessary.

## Discussion and conclusions

TSC is caused by the pathogenic variants in either the *TSC1* or *TSC2* tumor suppressor genes, located on chromosomes 9q34 and 16p13, respectively (3). *TSC1* and *TSC2* encode the protein hamartin and tuberin, which together suppress the activity of the mammalian target of rapamycin (mTOR) pathway (4). Loss of their function leads to continuous activation of the mTOR pathway, which results in cell proliferation (4). Typical clinical symptoms of TSC include periungual fibromas, renal angiomyolipoma, benign interstitial expansion of lung pulmonary smooth muscle cells, and neurological symptoms like seizures and neurodevelopmental delay (1).

Neurofibromatosis type 1 (NF1) is also an autosomal dominant genetic disorder caused by another tumor suppressor gene *NF1* (2). The *NF1* gene encodes neurofibromin, a negative regulator of the RAS–mitogen-activated protein kinase pathway. The loss of *NF1* contributes to the activation of this pathway and finally leads to tumor growth and development (5). Classic clinical features include café-au-lait macules, skinfold freckling, benign neurofibromas like cutaneous neurofibroma and plexiform neurofibroma, brain tumors, iris hamartomas, and typical bony lesions. Meanwhile, there are also reports of neurological manifestations such as epilepsy (6).

Comparing these two diseases, both may have vague and overlapping clinical presentations that can lead to missed diagnosis and finally cause delayed treatment. Coincidentally, both NF1 and TSC were first described by Von Recklinghausen, which proved from the side that these two diseases have some similar symptoms (7, 8). The major clinical diagnostic criteria of TSC such as hypomelanotic macule, angiofibroma, and shagreen patch are sometimes difficult to distinguish from typical NF1 phenomenon like café-au-lait macules, skinfold freckling, and benign neurofibroma (**Table 1**). Although NF1 and TSC are the first and second most common neurogenetic tumor syndromes, NF1 only has a prevalence of approximately 1:2,500 to 1:3,500, whereas TSC is even rarer and affects 1 in 5,500 to 1 in 10,000 live births (9). The limited amount of clinical cases further caused difficulties in clinical differential diagnosis, especially for doctors in remote areas. Moreover, it was shown that, in some cases, both NF1 and TSC could occur in a single individual, which might further confuse the diagnosis. We performed an extensive review of the literature using PubMed search including the words (NF1 and tuberous sclerosis) from the year 2000 until 2021 (**Table 2**) (10–14). Interestingly, Wheeler and Sadeghi-Nejad reported a case of a 4-month girl that suffered from both NF1 and TSC but reported a family history of NF1 only (12). After a definite diagnosis of TSC was made for this girl, her parents were carefully re-examined and also confirmed the diagnosis of TSC (12). This case further indicates the similarities of some clinical symptoms, and one definite diagnosis might further inhibit the recognition of another. Genetic testing and pathological biopsy may be necessary for a definite diagnosis of TSC. Moreover, as TSC and NF1 are both hereditary diseases, first-degree relatives should undergo a clinical assessment and a three-generation family history is required.

**Table 1:** Comparison of diagnosis criteria or neurofibromatosis type 1 and tuberous sclerosis complex.

	Neurofibromatosis type 1	Tuberous sclerosis complex
Major Features	1. Six or more café-au-lait macules (>5 mm in diameter in pre-pubertal children or >15mm in post-pubertal children); 2. Freckling in the axillary or groin; 3. Two or more neurofibromas of any type or one plexiform neurofibroma; 4. Optic glioma; 5. Two or more Lisch nodules; 6. A distinct osseous lesion such as sphenoid dysplasia; 7. A first-degree relative with neurofibromatosis type 1; 8. A pathogenic NF1 variant revealed by genetic testing.	1. Facial angiofibromas or forehead plaque; 2. Two or more non-traumatic ungual or periungual fibroma; 3. Three or more hypomelanotic macules (≥5 mm diameter); 4. Shagreen patch; 5. Three or more cortical dysplasias (includes tubers and perebral while matter radial migration lines); 6. Two or more subependymal nodules; 7. Two or more subependymal giant cell astrocytoma; 8. Multiple retinal nodular hamartomas; 9. Cardiac rhabdomyoma (single or multiple); 10. Lymphangioleiomyomatosis (LAM); 11. Angiomylipoma.
Minor Features	/	1. “Confetti” skin lesions; 2. Hamartomatous rectal polyps; 3. Gingival fibromas; 4. Pits in dental enamel; 5. Cerebral white matter radial “migration tracts”; 6. Retinal achromic patches; 7. Bone cysts.
Diagnosis Criteria	An individual must have two or more of these features.	Definite TSC: Two major features or a major feature with two minor features; probable TSC: one major feature and one minor feature; possible TSC: one major feature or two or more minor features.

**Table 2:** List of published case reports of patients with both NF-1 and TSC from 2000 to 2021.

Case (ref.)	Age (years)/sex	Publication year	TSC phenomena	NF1 phenomena	Therapy
1 (10)	15/female	2013	Facial angiofibroma; hypopigmented macule; shagreen patch; ungual fibroma	Café-au-lait macules; segmental freckling	None report
2 (11)	infant/female	2004	Hypopigmented macules; shagreen patch; family history	Café-au-lait macules; optic glioma; family history	Closely follow-up
3 (12)	infant/female	2005	Subependymal glial nodules; cutaneous hypopigmented patches; shagreen patch; family history; delayed cognitive development	Family history; Café-au-lait macules; right orbit superior (suspect of plexiform neurofibroma); delayed cognitive development	None report
4 (13)	20/female	2009	Shagreen patches; periungual fibroma; subependymal glial nodules; family history.	Café-au-lait macules; plexiform neurofibroma; axillary freckling	None report
5 (14)	24/male	2007	Seizure; shagreen patches; subependymal giant cell astrocytoma	Lisch nodules; Café-au-lait macules; plexiform neurofibroma	Right frontal craniotomy

Current clinical management of TSC is insufficient as most of them are symptomatic treatments, especially for seizures. Early therapeutic intervention for children who present with infantile spasms correlated with TSC is essential, and the first-line treatment is vigabatrin (15). Furthermore, the mTOR inhibitors such as everolimus have provided great therapeutic promise. A clinical study showed that treatment with everolimus in patients with TSC resulted in a sustained decrease in seizure frequency in children and adolescents (16). Nonetheless, this patient did not have any history of seizures. As he reached the age of 20 and presented with vascular blockage indicated by the non-healing ulcer, surgical approaches were considered according to the 2021 updated TSC international recommendations (17). DSA examination further showed signs of ischemia. As a result, amputation was the only surgical choice for him. Compared to the limited drug choices for patients with TSC, the development of drug therapies for patients with NF1 progressed rapidly. Selumetinib, a Mitogen-activated extracellularsignal-regulated kinase (MEK) inhibitor, was reported effective for plexiform neurofibroma and was approved for clinical usage by FDA (18). Early distinguishment of these two diseases is essential for further proper clinical treatment.

Another essential issue for the treatment of patients with TSC is life-long follow-up recommendations. TSC influences multiple organs in the body, and some manifestations are life-threatening. For example, our patient has lymphangioleiomyoma, which might cause respiratory dysfunction. According to the 2021 updated TSC international recommendations, we recommended routine serial pulmonary function test annually on chest CT (17). Meanwhile, we had several recommendations regarding other possible TSC features, including continuous sun protection for depigmented macules, detailed dental examination every 6 months, electrocardiography every 3 years for mild mitral regurgitation, annual ophthalmic evaluation, and renal function assessment. For patients with NF1, life-long follow-up is also essential, but they are less concerned with respiratory dysfunction. A definite diagnosis is essential for further proper follow-up.

Definite and accurate diagnosis is the basis for proper and effective treatment, and misdiagnosis might cause further damage to the patients and waste medical resources. It is far more essential for relatively rare diseases as most doctors might have relatively less experience with them. This case introduced the possible pitfall and similarities of clinical phenomena between TSC and NF1, which might be helpful for future clinical diagnosis and management of these diseases in this area.

## Data availability statement

The original contributions presented in the study are included in the article/**Supplementary Material**. Further inquiries can be directed to the corresponding authors.

## Ethics statement

The studies involving human participants were reviewed and approved by the institutional review board of Shanghai Ninth People’s Hospital, Shanghai Jiaotong University School of Medicine. The patients/participants provided their written informed consent to participate in this study. Written informed consent was obtained from the individual(s) for the publication of any potentially identifiable images or data included in this article.

## Author contributions

C-JW wrote the manuscript and was involved in the diagnostic and therapeutic clinical processes. L-LP analyzed radiology medical images and contributed to the diagnostic and therapeutic processes. M-HC made substantial contribution during revision. Z-CW and BW helped in the diagnostic process and critically revised the manuscript and were responsible for the diagnosis and treatment of the patient. All authors read and approved the final manuscript.

## Funding

This work was supported by grants from National Natural Science Foundation of China (82102344; 82172228; 81772115); Shanghai Rising Star Program supported by Science and Technology Commission of Shanghai Municipality (20QA1405600); Science and Technology Commission of Shanghai Municipality (19JC1413, 22MC1940300) ; Natural Science Foundation of Shanghai (22ZR1422300); “Chenguang Program” supported by Shanghai Education Development Foundation (SHEDF) (19CG18); Innovative research team of high-level local universities in Shanghai (SSMU-ZDCX20180700); the Project of Biobank (YBKA201901) from Shanghai Ninth People’s Hospital, Shanghai Jiao Tong University School of Medicine; Research Project of Multi-Disciplinary Team of Shanghai Ninth People’s Hospital (201907).

## Conflict of interest

The authors declare that the research was conducted in the absence of any commercial or financial relationships that could be construed as a potential conflict of interest.

## Publisher’s note

All claims expressed in this article are solely those of the authors and do not necessarily represent those of their affiliated organizations, or those of the publisher, the editors and the reviewers. Any product that may be evaluated in this article, or claim that may be made by its manufacturer, is not guaranteed or endorsed by the publisher.
